# Soft tissue handling and dental implant treatment

**DOI:** 10.1186/1746-160X-8-S1-I7

**Published:** 2012-05-25

**Authors:** Friedrich Wilhelm Neukam

**Affiliations:** 1 Department of Oral and Maxillofacial Surgery, University Hospital Erlangen, Germany

## 

Ectodermal dysplasias are a heterogeneous group of inherited disorders characterized by defective formation of tissues derived from the embryonic ectoderm. Besides tissues of epidermal origin, other maxillofacial structures including hard and soft tissues can be affected. Disturbances in the early development of teeth frequently result in congenital absence or deformities of teeth. The lack of teeth leads to an impaired development of the alveolar crest, with decreased vertical and horizontal dimension. Osseointegrated dental implants in combination with hard and soft tissue augmentation techniques can be used successfully to restore the patients’ masticatory function, phonetics and aesthetics.

